# How cholesterol regulates endothelial biomechanics

**DOI:** 10.3389/fphys.2012.00426

**Published:** 2012-11-15

**Authors:** Zhongkui Hong, Marius C. Staiculescu, Paul Hampel, Irena Levitan, Gabor Forgacs

**Affiliations:** ^1^Department of Physics and Astronomy, University of Missouri-ColumbiaColumbia, MO, USA; ^2^Pulmonary, Critical Care, and Sleep Medicine, Department of Medicine, University of Illinois at ChicagoChicago, IL, USA; ^3^Department of Biological Sciences, University of Missouri-ColumbiaColumbia, MO, USA

**Keywords:** tether force, cholesterol, atomic force microscopy (AFM), phosphatidylinositol-4,5-bisphosphate (PIP2)

## Abstract

As endothelial cells form the barrier between blood flow and surrounding tissue, many of their functions depend on mechanical integrity, in particular those of the plasma membrane. As component and organizer of the plasma membrane, cholesterol is a regulator of cellular mechanical properties. Disruption of cholesterol balance leads to impairment of endothelial functions and eventually to disease. The mechanical properties of the membrane are strongly affected by the cytoskeleton. As Phosphatidylinositol-4,5-bisphosphate (PIP2) is a key mediator between the membrane and cytoskeleton, it also affects cellular biomechanical properties. Typically, PIP2 is concentrated in cholesterol-rich microdomains, such as caveolae and lipid rafts, which are particularly abundant in the endothelial plasma membrane. We investigated the connection between cholesterol and PIP2 by extracting membrane tethers from bovine aortic endothelial cells (BAEC) at different cholesterol levels and PIP2 conditions. Our results suggest that in BAEC the role of PIP2, as a mediator of membrane-cytoskeleton adhesion, is regulated by cholesterol. Our findings confirm the specific role of cholesterol in endothelial cells and may have implications for cholesterol-dependent vascular pathologies.

## Introduction

The endothelium of blood vessels is a barrier that maintains the dynamic balance between blood flow and tissue. Disruption in endothelial cell function alters the permeability of this barrier and increases the risk of vascular disease, such as atherosclerosis, a potentially fatal abnormality of the circulatory system (Bonetti et al., 2003; Davignon and Ganz, 2004). Cholesterol is an essential component of the membrane of most cell types. Due to its association with cardiovascular disease, it plays particular role in endothelial cells. Alterations in the level of membrane cholesterol lead to significant changes in a number of cell functions, such as signaling (Parpal et al., 2001; Qin et al., 2006; Ramprasad et al., 2007), adhesion (Kaur et al., 2004; Qin et al., 2006; Ramprasad et al., 2007), motility (Vasanji et al., 2004; Qin et al., 2006), and remodeling of the cytoskeleton (Pierini et al., 2003; Riff et al., 2005; Qin et al., 2006; Iliev et al., 2007), as well as cell mechanical properties, such as fluidity (Brulet and McConnell, 1976; Cooper, 1978; Xu and London, 2000) and elasticity (Evans and Needham, 1986; Needham et al., 1988; Needham and Nunn, 1990). Elevation of membrane cholesterol level increases the stiffness of artificial lipid bilayers (el-Sayed et al., 1986; Needham and Nunn, 1990), whereas depletion in membrane cholesterol in the cellular plasma membrane makes cells typically less stiff (Vlahakis et al., 2002; Kwik et al., 2003). Surprisingly, the opposite result was found in endothelial cells: depletion in membrane cholesterol level strengthened the interaction between the plasma membrane and the cytoskeleton and resulted in the stiffening of endothelial cells (Byfield et al., 2004; Sun et al., 2007).

Decreasing cell stiffness upon depletion of membrane cholesterol could be attributed to the reduction in the level of phosphatidylinositol-4,5-bisphosphate (PIP2) in the plasma membrane (Kwik et al., 2003), as this molecule is known to be an important regulator of membrane-cytoskeleton association (Sechi and Wehland, 2000), and consequently of the cell's biomechanical properties (De Camilli et al., 1996; Rozelle et al., 2000; McLaughlin and Murray, 2005). Indeed, inactivation of PIP2 in fibroblasts resulted in decrease in membrane-cytoskeleton adhesion energy (Raucher et al., 2000).

The molecular mechanism underlying the cholesterol dependence of endothelial biomechanical properties is, however, not known. What is known is that cholesterol can induce the formation of special membrane microdomains, i.e., lipid rafts and caveolae (Anderson and Jacobson, 2002), particularly in endothelial cells (Zhu et al., 2003; Navarro et al., 2004), and these domains attract acidic lipids such as PIP2 (Pike and Casey, 1996; Pike and Miller, 1998; Sundaram et al., 2004). As depletion in the level of endothelial plasma membrane cholesterol leads to the strengthening of membrane-cytoskeleton adhesion, the role of PIP2 in endothelial biomechanics must be different from that in the other cell types studied so far (in particular fibroblasts). We asked what this role might be and how it is affected by changes in endothelial membrane cholesterol level.

We studied endothelial membrane biomechanics using atomic force microscopy (AFM)-based force spectroscopy to measure the force needed to extract membrane tethers (i.e., tether force) from bovine aortic endothelial cells (BAEC). In this technique the tether force *F*_*T*_ needed to pull a tether from the membrane at constant speed *v* is measured and the functional relation *F*_*T*_(*v*) is analyzed. For the analysis we used *F*_*T*_ = *F*_0_ + 2πη_*eff*_*v* (Hochmuth et al., 1996), where η_*eff*_ and *F*_0_ are, respectively, the membrane effective surface viscosity and the threshold pulling force. Effective surface viscosity means that η_*eff*_, in general, contains contributions associated with the intrinsic material properties of the lipid bilayer, the interbilayer slip and the connection of the membrane with the underlying cytoskeleton (Hochmuth et al., 1996). The force *F*_0_ is a direct measure of the membrane-cytoskeleton adhesion energy and membrane stiffness (Hochmuth et al., 1996; Sheetz, 2001). The above relationship has been widely employed to quantify the results of tether-pulling experiments (Hochmuth et al., 1996; Shao and Hochmuth, 1996; Hochmuth and Marcus, 2002; Girdhar and Shao, 2007; Liu et al., 2007; Sun et al., 2007). In particular it allowed for an accurate quantitative description of our earlier AFM studies on endothelial biomechanics (Sun et al., 2007).

The above-described method has extensively been used to quantitatively assess the strength of membrane-cytoskeleton association (Sun et al., 2005, 2007; Cuerrier et al., 2009). To establish the role of PIP2 in endothelial membrane-cytoskeleton interaction, we investigated the localization and distribution of PIP2 in BAEC and its effect on the tether force under variable PIP2 and cholesterol conditions. Our results indicate that in endothelial cells PIP2 functions in a cholesterol-dependent manner, in a way that is different or even opposite to that observed in other cell types. Our experiments also elucidate the mechanism of PIP2 regulation by cholesterol. These results are consistent with the particular role of cholesterol in endothelial biomechanics.

## Materials and methods

### Cell culture and cholesterol depletion

BAEC between passages 15 and 18 were cultured in Dulbecco's Modified Eagle's Medium (DMEM; Cell Grow, Washington, DC) supplemented with 10% Fatal Bovine Serum (FBS; ATALANTA Biologicals, Lawrenceville, GA). Human skin fibroblasts (HSFs; CRL-2522; American Type Culture Collection (ATCC), Manassas, VA) were grown in DMEM containing 20% F-12 Nutrient mixture (Ham) (Life Technologies, Grand Island, NY) and 20% FBS. Cell cultures were maintained in a humidified incubator at 37°C and 5% CO_2_. The cells were fed and split every 2–3 days. For cholesterol depletion, BAEC were incubated in 5 mM methyl-β-cyclodextrin (MβCD, Sigma, St. Louis, MO) in serum free medium for 45 min (Byfield et al., 2004).

### PH-PLC-GFP transfection

Inactivation of PIP2 was accomplished by transfecting HSFs and BAEC with PH-PLC-GFP (i.e., GFP tagged plextrin homology domain of phospholipase C). PH-PLC-GFP inactivates PIP2 through sequestration. For PH-PLC transfection, 60–70% confluent HSFs and BAEC on 18 mm square glass cover slips were washed with 2 ml OPTI-MEM® I Serum Free Medium (Invitrogen, Grand Island, NY). Next, cells were incubated at 37°C, 5% CO_2_ for 4 h in 1 ml OPTI-MEM® I Reduced Serum Medium supplemented with 4 μg tris-EDTA buffer (TE) diluted DNA plasmid vector that contained the PC-PLC-GFP construct and 4 μl Lipofectamine (Invitrogen, Grand island, NY). Finally, the transfection medium was gently removed and replaced with 2 ml CO_2_-independent DMEM with 2% FBS. For comparison, BAEC were also transfected with only GFP, following the above procedure. Images of BAEC were acquired 24 h post-transfection with fluorescent PH-PLC-GFP, using a Bio-Rad Radiance 2000 confocal system (Carl Zeiss Microimaging, Thornwood, NY).

### Delivery of exogenous PIP2 into endothelial cells

Exogenous PIP2 was delivered into BAEC by Echelon PIP2-Shuttle kit (Echelon Biosciences, Inc. Salt Lake City, UT). Cells were washed twice with serum free DMEM, followed by incubation at 37°C for 30 min in serum free DMEM containing 10 μM PIP2-Shuttle complex (Wang et al., 2003). For the combination of cholesterol depletion and exogenous PIP2 enrichment, cholesterol depletion was performed first.

### Measurement of tether force

Measurements were performed at room temperature as described earlier (Sun et al., 2005). Briefly, a 60-mm Petri dish containing cells in CO_2_-independent medium with 2% FBS, 10 μg/ml penicillin, streptomycin, and kanamycin sulphate was placed under AFM. The cantilever was lowered toward the cell until contact with the cell membrane was established. Contact was maintained for 8 sec, after which the cantilever was retracted at a constant pulling speed (3, 9, 15, and 21 μm/s). The tether force was identified with the width of the steps of the discontinuities in the force-elongation curve. Several hundred step-like events were recorded using 20–30 cells, with each cell subjected to multiple pulling experiments. Data analysis was carried out with Igor Pro 6 (WaveMetrics, Lake Oswego, OR) and Origin Pro 8 (OriginLab Corporation, Northampton, MA).

### Fluorescent PIP2 imaging

Fluorescent images were acquired using a Bio-Rad Radiance 2000 confocal system (Carl Zeiss Microimaging, Thornwood, NY). Cell shape and distribution of fluorescent PIP2 as function of cholesterol treatment was also visualized with Differential Interference Contrast (DIC) microscopy. Images were acquired using the DIC imaging module on the Zeiss LSM 510 Meta NLO 2-photon confocal (Carl Zeiss, Thornwood, NY) with a 60X 1.4 NA Zeiss Plan Apochromat water immersion objective.

### Statistical analysis

Data were expressed as mean ± standard error of the mean (SEM). Analysis of covariance was performed to compare the results of tether-pulling experiments under different conditions using OriginPro 8.0 (OriginLab Corporation, Northampton, MA). A value of *P* < 0.05 was used to establish statistical significance.

## Results

### Localization of PIP2

**Figure 1:**
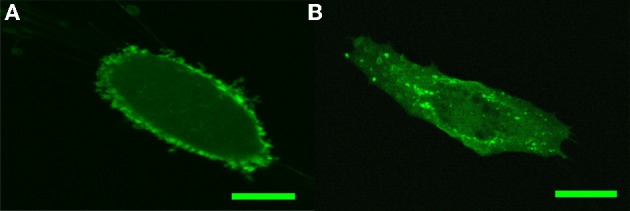
**Confocal imaging of PH-PLC-GFP expression pattern in HSF and BAEC. (A)** Characteristic PH-PLC-GFP expression in HSF. **(B)** Characteristic PH-PLC-GFP expression in BAEC. Scale bars: 20 μm.

PIP2 intracellular distribution in fibroblasts and endothelial cells was visualized by transfecting the cells with PH-PLC-GFP construct known to bind specifically to PIP2. In earlier studies on fibroblasts it was found that PH-PLC-GFP is strongly and uniformly localized along the plasma membrane (Raucher et al., 2000). As shown in Figure 1A, our own measurements on HSF support these earlier findings. This pattern is consistent with the ability of PH-PLC-GFP to sequester PIP2 and the latter's involvement in the linkage of the membrane with the cortical actin cytoskeleton. As Figure 1B indicates, this is in contrast with our findings in PH-PLC-GFP-expressing BAEC: strong localization around the nucleus, punctate distribution along the cell membrane and homogeneous dispersion throughout the cell.

### Sequestration of PIP2 does not affect the tether force in BAEC

**Figure 2:**
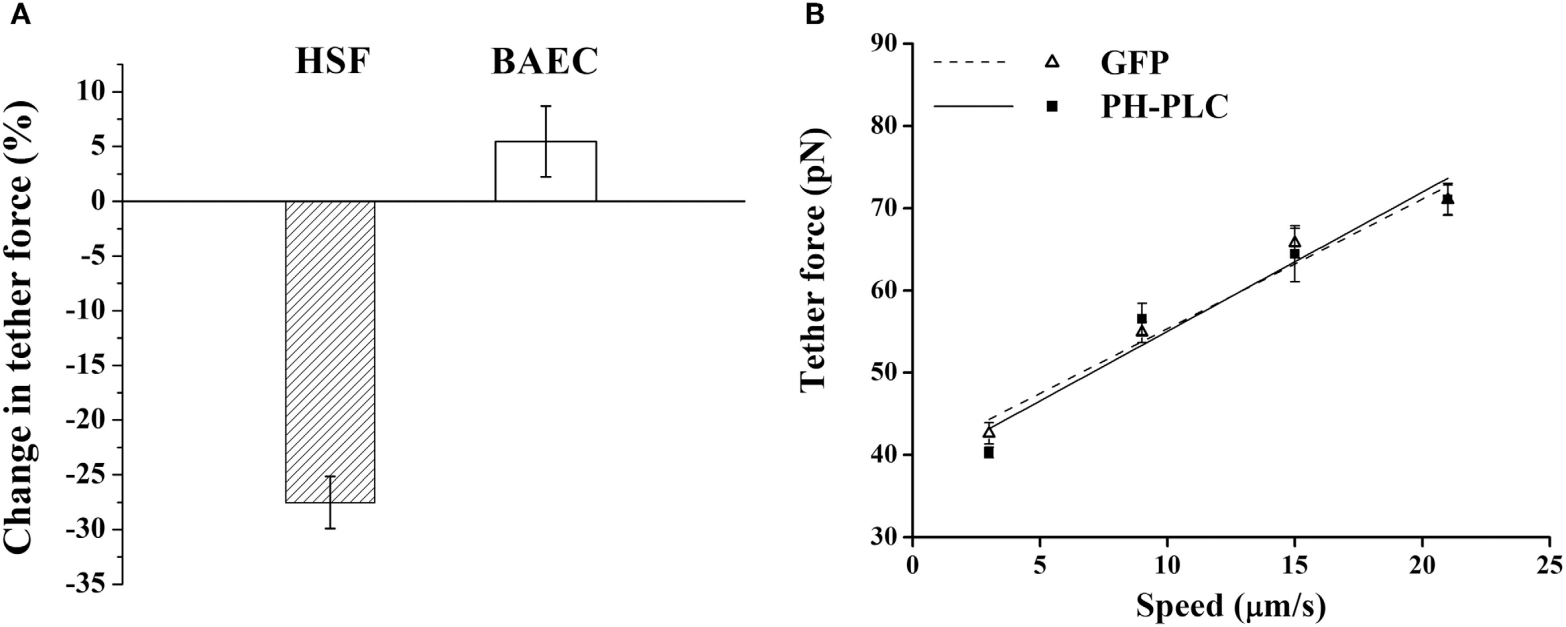
**Effect of PIP2 on the tether force. (A)** Change in the magnitude of tether force upon PH-PLC-GFP transfection at 15 μm/s tether extraction speed. In HSF the negative value of the change corresponds to a drop in tether force, whereas in BAEC the change is within the experimental error. **(B)** Tether force versus pulling speed in BAEC transfected with PH-PLC-GFP. Cells treated with GFP alone were used as control. The two curves are statistically indistinguishable (*p* > 0.05). (The slope of two curves, 2πη_*eff*_, is 1.60 pN·s/μm and 1.67 pN·s/μm, respectively, for the GFP treated cells and cells transfected with PH-PLC-GFP). Error bars represent SEM.

Next, we compared how membrane-cytoskeleton adhesion is affected by changes in PIP2 in BAEC with PH-PLC-GFP expression. For this we used AFM force spectroscopy to measure the magnitude of the tether force needed to extract membrane tethers from the cell. Whereas our own measurements on HSF supported the earlier observation that expression of PH-PLC in fibroblasts leads to a considerable drop in the magnitude of the tether force (Raucher et al., 2000; Figure 2A), we found contrasting results in BAEC. Expression of PH-PLC-GFP in BAEC did not alter the tether force (Figure 2B). This finding is inconsistent with the role of PIP2 being a direct regulator of membrane-cytoskeleton cross-linking in BAEC.

### Increase in PIP2 level does not alter membrane-cytoskeleton adhesion in BAEC

**Figure 3:**
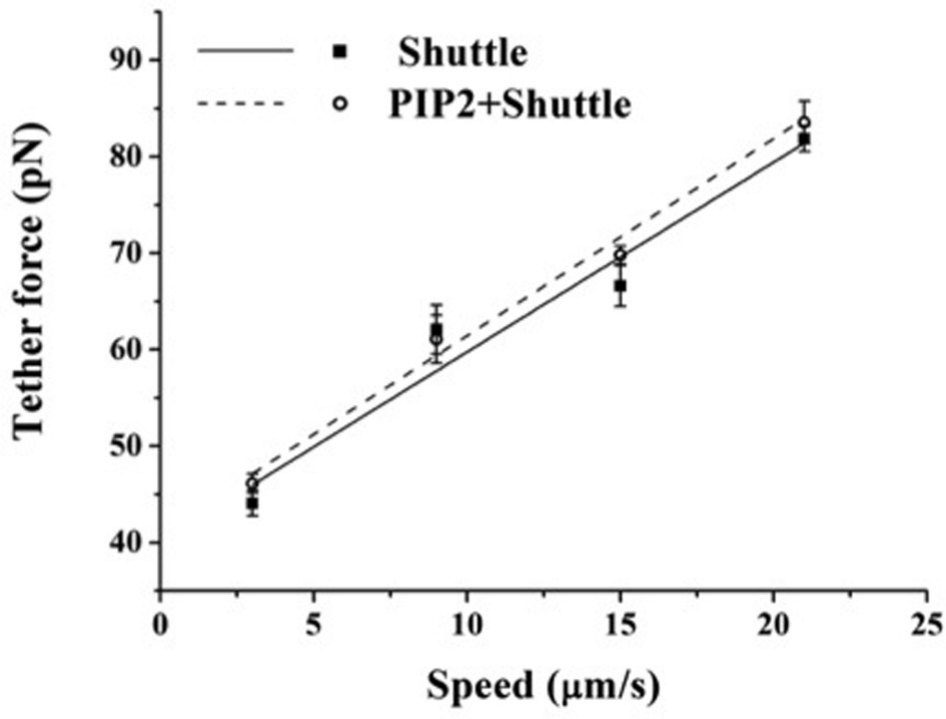
**Effect of exogenous PIP2 on the tether force.** Tether force vs. pulling speed in BAEC treated with PIP2-Shuttle. Cells treated with the Shuttle alone were used as control. The two curves are statistically indistinguishable (*p* > 0.05). (The slope of the curves, 2πη_*eff*_, is 1.97 pN·s/μm and 2.04 pN·s/μm, respectively, for the cells treated with Shuttle alone and cells loaded with exogenous PIP2+Shuttle). Error bars represent SEM.

If PIP2 acts as a membrane-cytoskeleton cross-linker, increase in its cellular level is expected to result in the increase in membrane-cytoskeleton adhesion and thus in the tether force. However, upon exogenously increasing the PIP2 level in BAEC (with the PIP2-shuttle kit) we found no change in tether force (Figure 3).

### Cholesterol controls the distribution of PIP2 in BAEC

**Figure 4:**
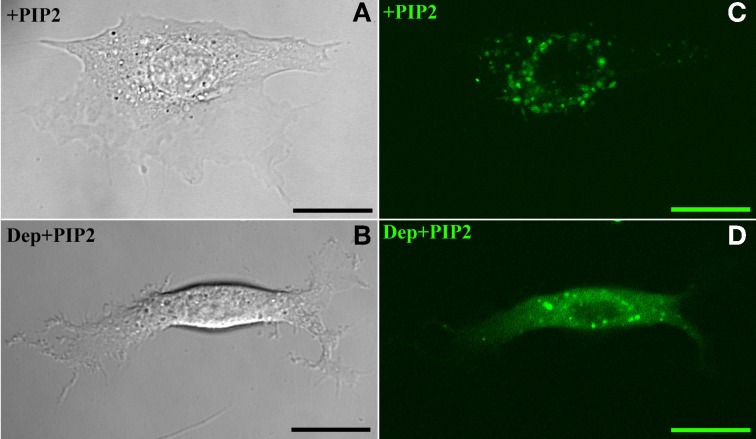
**Exogenous PIP2 localization.** Panels on the left and right, respectively, refer to images acquired with Differential Interference Contrast and confocal microscopy. **(A,C)** PIP2-loaded BAEC. **(B,D)** Cholesterol-depleted BAEC loaded with exogenous PIP2. Scale bars: 20 μm.

**Figure 5:**
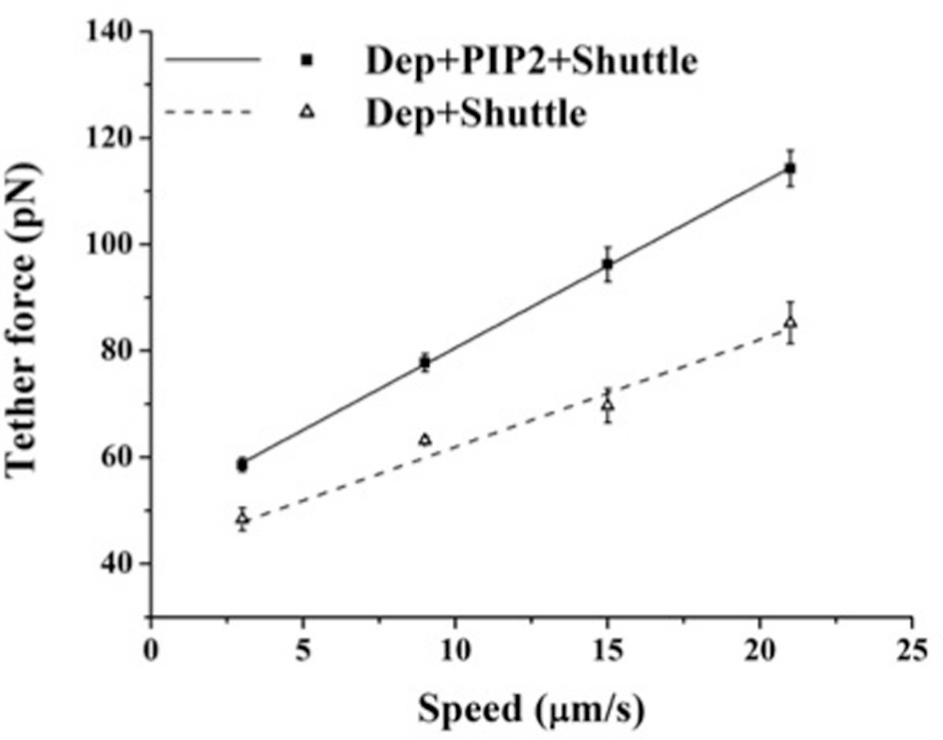
**Effect of exogenous PIP2 on the tether force upon cholesterol depletion.** Tether force versus pulling speed in BAEC treated with MβCD followed with the delivery of PIP2-Shuttle or only Shuttle. The two curves are statistically different (*p* < 0.05). (Values of the slopes are given in the text). Error bars represent SEM.

The above findings suggest that in BAEC membrane-cytoskeleton interactions are insensitive to changes in PIP2 level. On the other hand earlier work pointed to the peculiar role of cholesterol in the modulation of endothelial membrane-actin cytoskeleton association (Sun et al., 2007). To clarify how these observations may possibly be compatible with each other we subjected BAEC to membrane cholesterol depletion (with MβCD) followed by delivery of exogenous PIP2. Cholesterol depletion resulted in drastic changes in the localization of fluorescent PIP2. Along with the decrease in the number of discrete patches (as can be visually observed by comparing Figures 4C,D), a more uniform distribution across the cell appeared. In addition, the measured tether force in BAEC depleted in cholesterol and enriched with exogenous PIP2 was significantly larger than in cells depleted in cholesterol and enriched with shuttle alone (Figure 5). The difference can be quantified in terms of the slopes of the curves in Figure 5, which provide the values of η_*eff*_. The slope of two curves (2πη_*eff*_) is 3.08 pN·s/μm and 2.02 pN·s/μm, respectively, for the Dep+PIP2+Shuttle and the Dep+Shuttle.

## Discussion

A major objective of this work was to elucidate the role of PIP2, as a membrane-cytoskeleton cross-linker in endothelial cells. This objective was motivated by earlier findings on the consequences of cholesterol depletion of the plasma membrane, leading, respectively, to the increase and decrease of membrane-cytoskeleton adhesion in BAEC (Sun et al., 2007) and fibroblasts (Kwik et al., 2003). Since the effect of cholesterol depletion in fibroblasts is mimicked by inactivation of PIP2 (Kwik et al., 2003) (both leading to decrease in membrane-cytoskeleton adhesion), we sought the molecular basis of cholesterol depletion-induced stiffening of BAEC also in the mechanism of PIP2 action. For this end, we first investigated the cellular distribution of PIP2 in endothelial cells by transfecting BAEC with PH-PLC-GFP, a PIP2 sequestering agent, and observing its expression pattern in the cell. The PH-PLC-GFP expression pattern was found to significantly differ from that in HSF. In particular, instead of the uniform expression along the cell membrane in HSF (Figure 1A), in BAEC a more punctate pattern was found (Figure 1B). In addition, whereas in HSF PIP2 sequestration led to the decrease in membrane-cytoskeleton adhesion energy [Figure 2A (Raucher et al., 2000)], in BAEC it resulted in no change (Figures 2A,B).

Next, we considered the effect of exogenous PIP2. If PIP2 is a regulator of membrane-cytoskeleton cross-linking then it is to be expected that loading the cell with PIP2 would result in the increase in this cross-linking. Contrary to this expectation we found no change, as assessed by measuring the tether force in PIP2-loaded BAEC (Figure 3).

The above results suggest that whereas PIP2 is an important membrane-cytoskeleton cross-linker in fibroblasts, it does not act as such in endothelial cells: neither decrease (via sequestration) or increase (via loading) in PIP2 concentration led to any detectable change in the tether force in BAEC.

Motivated by earlier findings on the particular role of cholesterol in BAEC (Byfield et al., 2004; Sun et al., 2007), we then considered the effect of cholesterol depletion on the properties of PIP2 in BAEC. Cholesterol depletion led to the uniform distribution of exogenous PIP2 (Figure 4) and to the re-establishment of PIP2's function as a membrane-cytoskeleton cross-linker, again assessed by measurement of the tether force (Figure 5).

**Figure 6:**
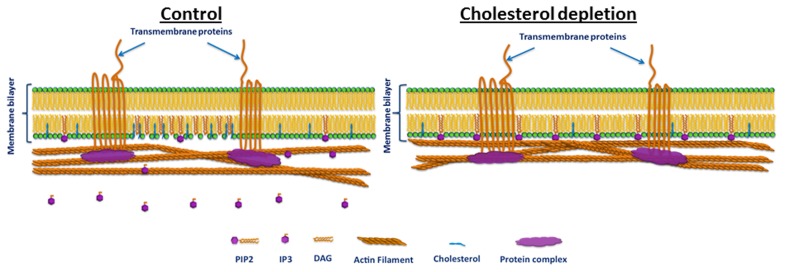
**Schematic illustration of the hypothesized cholesterol regulation of PIP2 function in BAEC. Left:** cholesterol induces the formation of low-density lipid plasma membrane microdomains, such as caveolae, and facilitates PIP2 hydrolysis and thus its loss of cross-linker function. **Right:** cholesterol depletion destructs caveolae, thus reduces PIP2 hydrolysis and restores PIP2's cross-linker function. Note that membrane-cytoskeleton attachment via PIP2 is typically complementary to other mechanisms such as those that involve transmembrane proteins (e.g., cadherins, which couple to the F actin network through protein complexes). Thus, even when the PIP2 pathway is abrogated the membrane and the cytoskeleton may remain linked.

The presented results provide strong indication that in endothelial cells the role of PIP2 as a membrane-cytoskeleton cross-linker is regulated by cholesterol. Furthermore, they confirm the findings of earlier studies on the special role of cholesterol in the regulation of endothelial cell biomechanical properties. They point to a possible specific molecular mechanism for this regulation (presented schematically in Figure 6). The hypothesized mechanism is consistent with known information on the role of cholesterol in the formation and maintenance of caveolae and the localization of PIP2, and cholesterol depletion leading to the inhibition of PIP2 turnover in these membrane microdomains (Pike and Casey, 1996; Pike and Miller, 1998; Sundaram et al., 2004). Accordingly, we hypothesize that cholesterol induces the formation of caveolae in BAEC and facilitates the hydrolysis of PIP2 into inositol 1,4,5-trisphosphate (IP3) and diacylglycerol (DAG) resulting in the loss of PIP2's function as a membrane-cytoskeleton cross-linker. Cholesterol depletion leads to the partial destruction of caveolae, thus to the partial inhibition of PIP2 hydrolysis. This in turn results in the reinforcement of PIP2's role as a membrane-cytoskeleton cross-linker (Figure 6). The results of this work may offer valuable information for studies on cholesterol-dependent vascular pathologies, such as atherosclerosis, that affect endothelial biomechanical properties in general and the endothelial plasma membrane in particular.

### Conflict of interest statement

The authors declare that the research was conducted in the absence of any commercial or financial relationships that could be construed as a potential conflict of interest.
